# Supplementary material for: Electric control of a canted-antiferromagnetic Chern insulator
Source: Nat Commun. 2022 Mar 29;13:1668. doi: 10.1038/s41467-022-29259-8 (PMC8964814; doi:10.1038/s41467-022-29259-8)
Supplement: Supplementary file 1 — Supplementary Information [file 41467_2022_29259_MOESM1_ESM.pdf]

Title: Electric control of a canted-antiferromagnetic Chern insulator

Authors: Jiaqi Cai[1*], Dmitry Ovchinnikov[1*], Zaiyao Fei[1], Minhao He[1], Tiancheng Song[1], Zhong Lin[1], Chong Wang[1,2], David Cobden[1], Jiun-Haw Chu[1], Yong-Tao Cui[3], Cui-Zu Chang[4], Di Xiao[1,2], Jiaqiang Yan[5], Xiaodong Xu[1,2§]

Affiliations:
[1]Department of Physics, University of Washington, Seattle, Washington 98195, USA
[2]Department of Materials Science and Engineering, University of Washington, Seattle, Washington 98195, USA.
[3]Department of Physics and Astronomy, University of California, Riverside, California 92521, USA
[4]Department of Physics, The Pennsylvania State University, University Park, Pennsylvania 16802, USA
[5]Materials Science and Technology Division, Oak Ridge National Laboratory, Oak Ridge, Tennessee 37831, USA.
*These authors contributed equally to the work.
[§]Correspondence to xuxd@uw.edu

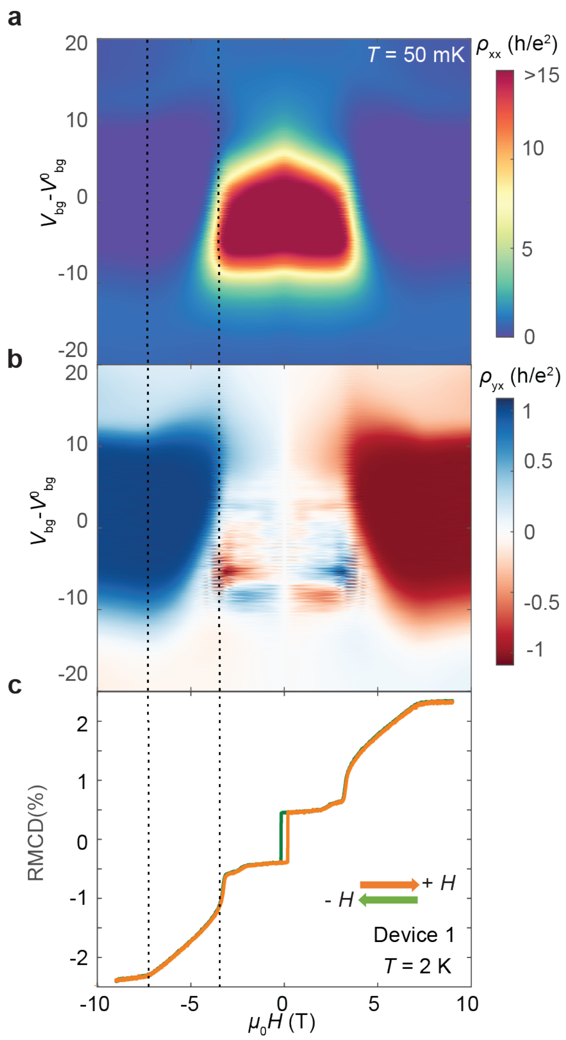

Supplementary Figure 1. Gate dependent magneto-transport and RMCD characterization of Device 1. a-b, ρ_{xx} (**a**) and ρ_{yx} (**b**) as a function of $\mu_0 H$ and V_{bg}-V_{bg}^0 where V_{bg}^0 =44 V. **c**, RMCD signal adopted from Fig. 1 in the Maintext. The black dashed lines denote where C=1 Chern insulator states in the canted-AFM and field-induced FM appear in the dual gated map (**a-b**).

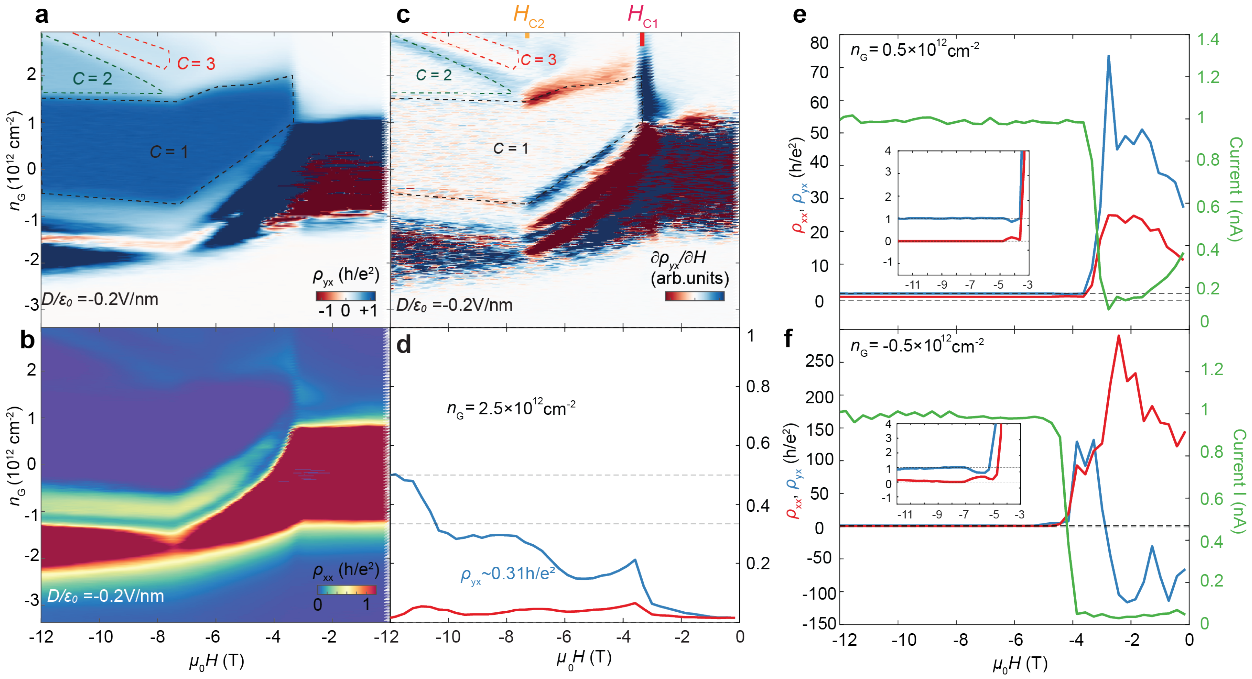

Supplementary Figure 2. Magneto-transport data of Device 2. **a,b,** ρ_{yx} and ρ_{xx} as a function of n_G and $\mu_0 H$ at D/ε_0 = -0.2 V/nm. The dashed lines are contours defined by ρ_{yx} = (0.97, 0.47, 0.28) h/e^2, corresponding to the C = 1,2,3 states. **c,** The derivative of data in panel (**a**) with respect to magnetic field. **d-f,** The horizontal linecut of ρ_{xx} in red, ρ_{yx} in blue at n_G of **(d)** 2.5×10^{12} cm^{-2}, **(e)** 0.5×10^{12} cm^{-2}, and **(f)** -0.5×10^{12} cm^{-2}**.** Insets in **(e) and (f)** are the zoom in plot of ρ_{xx} and ρ_{yx} at high magnetic fields. The excitation current is also shown in green for (**e-f**). In the AFM state, the sudden drop of the current highlights a highly resistive state with respect to 100MΩ resistor in series (see Methods) in low field part. This large resistance also leads to an overload feature of ρ_{yx}.

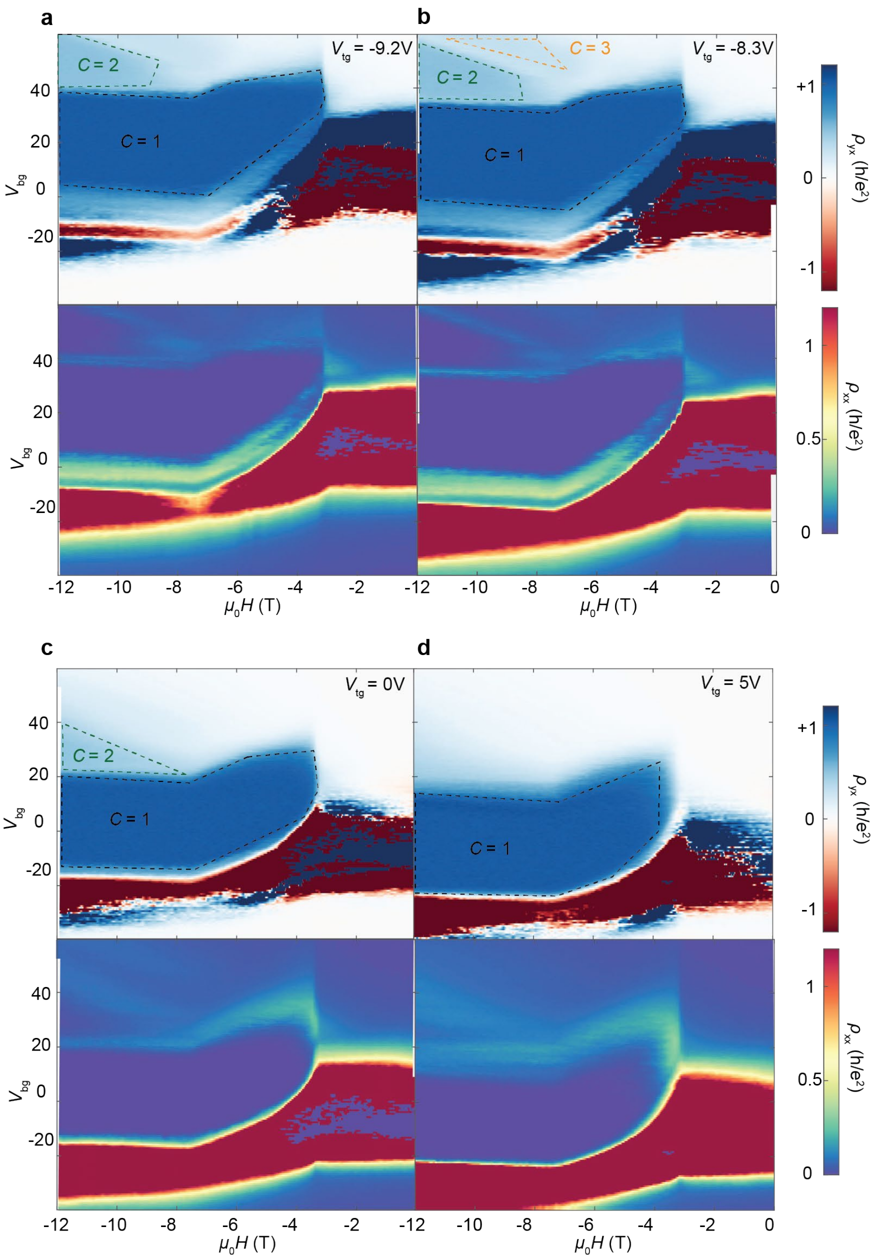

Supplementary Figure 3. Top gate-controlled topological phase diagram of Device 2. a-d, ρ_{yx} (top) and ρ_{xx} (bottom) as a function of $\mu_0 H$ and back gate voltage V_{bg} at selected top gate voltages, V_{tg} = -9.2 V(**a**), -8.3V (**b**), 0V (**c**), and 5V (**d**). The dashed lines are contours defined by ρ_{yx} = (0.97, 0.47, 0.28) h/e^2 indicating the C = 1, 2, 3 states. The C = 2 state only appears in high top gate voltages (**a-c**), corresponding to negative electric field at given positive V_{bg}. It is visible that tuning the top gate changes quantization field from -3.3 T to -4 T in the phase diagram.

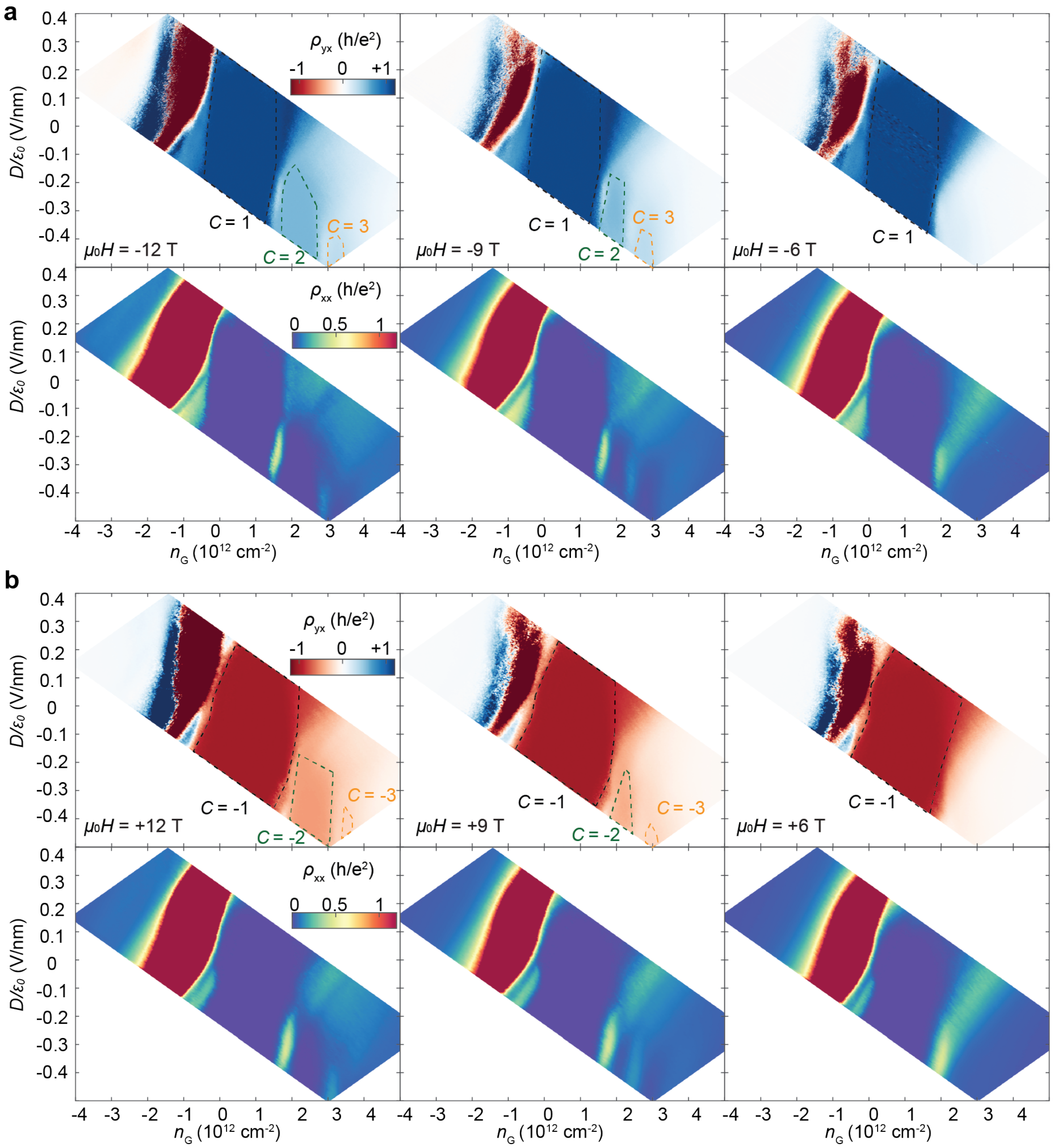

Supplementary Figure 4. Magnetic field dependent transport maps for Device 2. a-b, ρ_{yx} (top) and ρ_{xx} (bottom) as a function of D/ε_0 and n_G at $\mu_0 H$ = -12 T, -9 T and -6 T (**a**); $\mu_0 H$ = +12 T, +9 T and +6 T (**b**). Dashed lines enclose the topological phases with different Chern numbers, defined by the contours of $\rho_{yx} = \pm(0.97,\ 0.47,\ 0.28)\ h/e^2$. We observed a strong electron and hole asymmetry as the electronic bands of $MnBi_2Te_4$ have an electron like topological number ($\rho_{yx} = -\mathrm{sgn}(H)h/e^2$). When the first Landau level with $\nu = -1$ of the hole band develops, the hole band has a Chern number $C = 1 + (-1) = 0$ and possibly develops an insulating state. There is also electron-hole asymmetry in $MnBi_2Te_4$'s band structure.

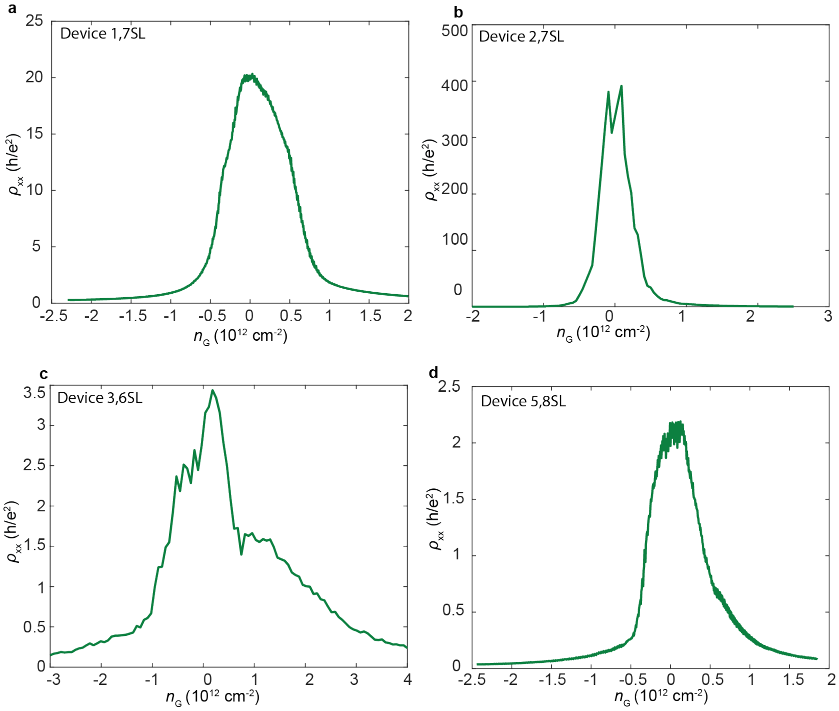

Supplementary Figure 5. Longitudinal resistance (ρ_{xx}) of all devices at zero magnetic field. a-d, ρ_{xx} as the function of gate-induced carrier density n_G. For dual-gated devices (**b-c**, Device 2 and 3), the electric field is fixed at $D/\varepsilon_0 = 0$ by simultaneously sweeping both gates. For single gated device, a back gate is used to tune the carrier density. All data are taken at the temperature $T = 50$mK.

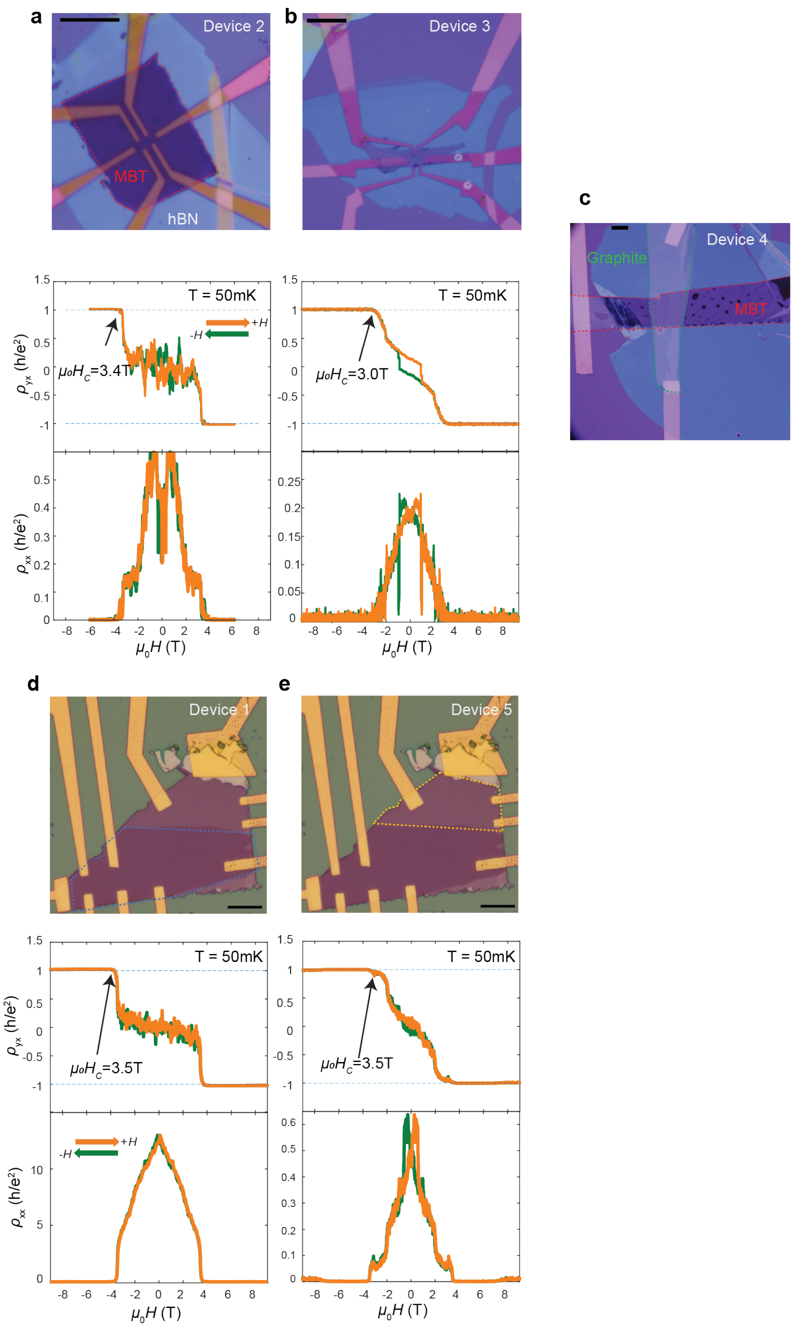

Supplementary Figure 6. Optical microscope images of all devices and basic magneto-transport characterizations. a-b, Optical microscope images of dual-gated devices (top), and corresponding ρ_{yx} (middle) and ρ_{xx} (bottom) as function of $\mu_0 H$. The untransparent gold top gate is not shown for clarity. **c,** Optical microscope image of Device 4, a dual gated device for optical investigation. **d-e,** Optical microscope image of single gated devices (top), and corresponding ρ_{yx} (middle) and ρ_{xx} (bottom) as function of $\mu_0 H$. Devices 1 and 5 are two parts of the same flake, enclosed by blue (Device 1) and yellow (Device 5) dashed lines. The $\mu_0 H_C$ is extracted at ρ_{yx}~$0.98h/e^2$. Scale bar: 10 μm.

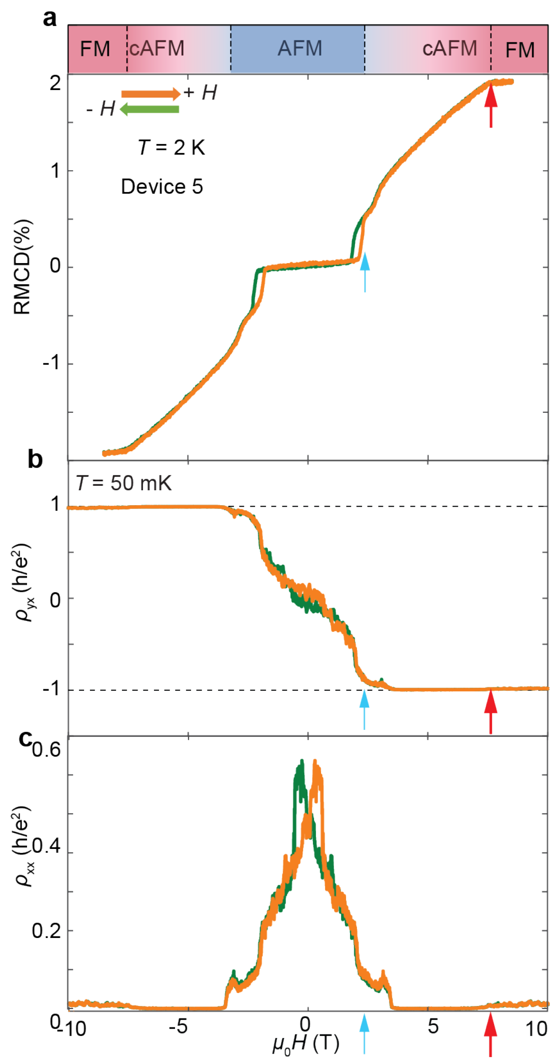

Supplementary Figure 7. RMCD and transport data in an additional single gated device (Device 5). a, RMCD signal taken at a temperature of 2 K at V_{bg} = 0 V. **b,** ρ_{yx}, and **c,** ρ_{xx} measurements as a function of magnetic field ($\mu_0 H$) at optimal gate voltage V_{bg} = 42 V.

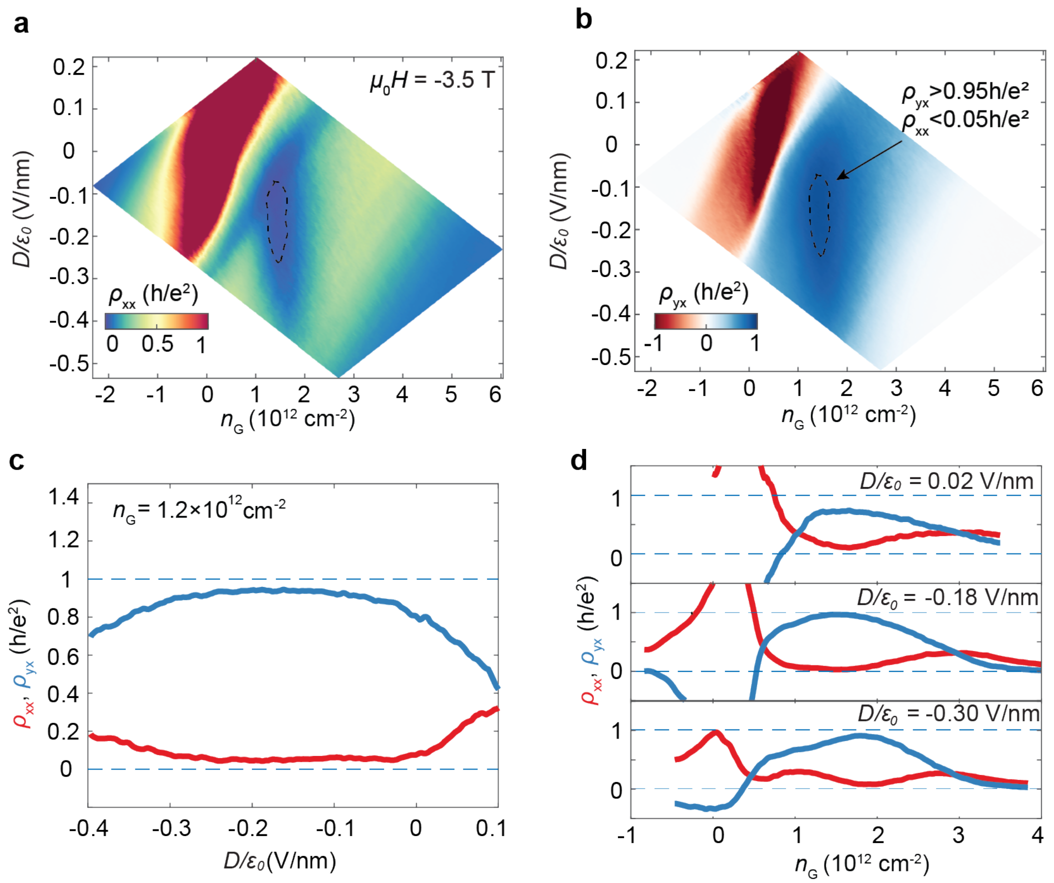

Supplementary Figure 8. Electric tuning of canted-AFM Chern insulating state for Device 2. a, ρ_{xx}, **b,** ρ_{yx} as a function of D/ε_0 and n_G at fixed magnetic field $\mu_0 H$ = -3.5 T. The dashed lines are contours with ρ_{xx}<0.05 h/e^2 and ρ_{yx}>0.95 h/e^2, indicating the phase space of the C = 1 state. **c** ρ_{xx} (red) and ρ_{yx} (blue) as a function of D/ε_0 at n_G =1.2×10^{12} cm^{-2}, which are vertical linecuts from **a** and **b**. **d,** n_G dependent ρ_{xx} and ρ_{yx} at selected D/ε_0, which are horizontal linecuts from **a** and **b**.

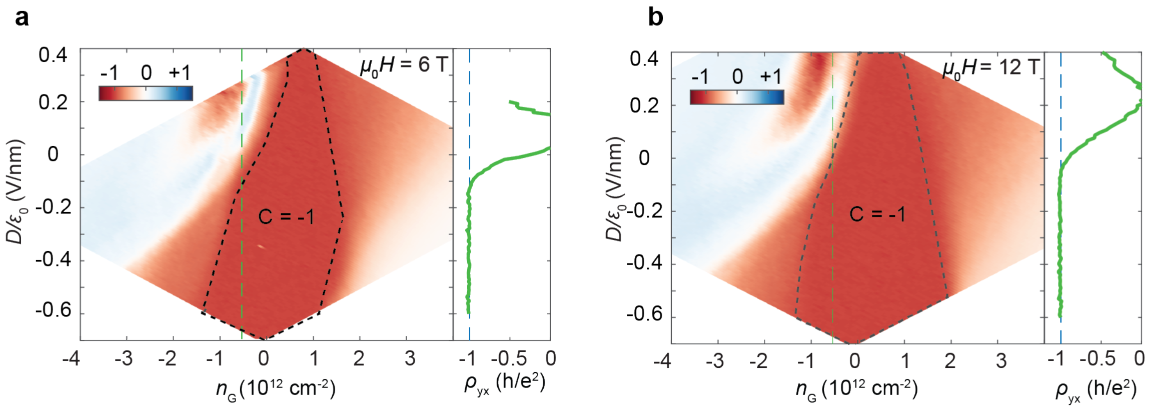

Supplementary Figure 9. Electrical tuning of the Chern insulator state away from spin-flop fields for Device 3. a,b, ρ_{yx} as a function of electric field D/ε_0 and gate induced carrier density n_G at fixed $\mu_0 H$ of 6 T (**a**), and 12 T (**b**). The side panels in **a** and **b** are linecuts from the main panels along the green dashed lines at n_G = - 0.5×10^{12} cm^{-2}, which is away from optimal doping. Near optimal carrier doping, the electric field effect on ρ_{yx} is marginal. The black dashed lines are contours with ρ_{yx}>0.97 h/e^2, indicating the phase space of the C = 1 state.

Device type	Device # in the text	Thickness (# of SLs)	cAFM Chern insulator?	Mobility (10^3cm^{2}V^{-1}s^{-1}) (50mK, ~3×10^{12}cm^{-2})
Single gated device	1	7	Y	0.2
	5	8	Y	0.9
Dual gated device	2	7	Y	1.7-3
	3	6	Y	1.5-2

Supplementary Table 1. A list of the transport devices. To obtain electron mobility, we gate the device to be far away from anomalous Hall region and extract Hall carrier density and mobility by fitting the linear Hall effect with respect to small magnetic field.
